# Model-Based Policymaking: A Framework to Promote Ethical “Good Practice” in Mathematical Modeling for Public Health Policymaking

**DOI:** 10.3389/fpubh.2017.00068

**Published:** 2017-04-05

**Authors:** Lisa A. Boden, Iain J. McKendrick

**Affiliations:** ^1^School of Veterinary Medicine, College of Medical, Veterinary and Life Sciences, University of Glasgow, Glasgow, UK; ^2^Biomathematics and Statistics Scotland, JCMB, The King’s Buildings, Edinburgh, UK

**Keywords:** mathematical models, ethics, policymaking, justice, independence, beneficence, transparency

## Abstract

Mathematical models are increasingly relied upon as decision support tools, which estimate risks and generate recommendations to underpin public health policies. However, there are no formal agreements about what constitutes professional competencies or duties in mathematical modeling for public health. In this article, we propose a framework to evaluate whether mathematical models that assess human and animal disease risks and control strategies meet standards consistent with ethical “good practice” and are thus “fit for purpose” as evidence in support of policy. This framework is derived from principles of biomedical ethics: independence, transparency (autonomy), beneficence/non-maleficence, and justice. We identify ethical risks associated with model development and implementation and consider the extent to which scientists are accountable for the translation and communication of model results to policymakers so that the strengths and weaknesses of the scientific evidence base and any socioeconomic and ethical impacts of biased or uncertain predictions are clearly understood. We propose principles to operationalize a framework for ethically sound model development and risk communication between scientists and policymakers. These include the creation of science–policy partnerships to mutually define policy questions and communicate results; development of harmonized international standards for model development; and data stewardship and improvement of the traceability and transparency of models *via* a searchable archive of policy-relevant models. Finally, we suggest that bespoke ethical advisory groups, with relevant expertise and access to these resources, would be beneficial as a bridge between science and policy, advising modelers of potential ethical risks and providing overview of the translation of modeling advice into policy.

## Introduction

Access to “big data” and new computing technologies has increased the utility of mathematical models and algorithms, including the potential to “fast-track” decision-making to mitigate risks. They enable scientists to explore experimental hypotheses that are difficult or unethical to test in complex real-world systems. They have also been used as forecasting tools to assess disease risk, improve surveillance, understand the implications of risk-mitigating interventions, and allocate resources during public health emergencies (1, 2).

Many epidemiological forecasting models have utilitarian aims: to minimize risk of disease and to maximize health for the study population. Although the modeling process may diminish uncertainties about disease risks, it may also uncover or create new ethical problems that have impacts upon risk management. The foreseeable, predictable, yet uncertain nature of these issues and an absence of ownership or accountability for the management of ethical risk (as a joint science-policy problem), creates a moral dilemma that needs to be resolved before models, as evidence for policy, can be considered truly “fit for purpose.”

In this article, we propose a framework based on principles of biomedical ethics to evaluate whether mathematical models that assess human and animal disease risks and control strategies meet “good practice” standards and are “fit for purpose” as evidence in support of policy. We subsequently propose initiatives to improve opportunities for ethical model building and risk communication between scientists and policymakers.

## Framework to Promote Ethical, “Good Practice” Standards

Although there are informal guidelines that specify best practice, there are no formal agreements about what constitutes professional competencies or duties in mathematical modeling for public health [(3, 4), p. 18]. Thus, it is difficult to objectively assess whether models produce policy evidence that is “fit for purpose,” and it is unclear to whom this responsibility belongs (i.e., policymaker or scientist). Nonetheless, if model evidence is not assessed objectively, there is a possibility that ethical mistakes may be made that ultimately undermine the success of policies and regulatory decisions in human and animal health and erode public trust in science and government.

We propose four major criteria that offer a framework to determine the suitability of scientific outputs as evidence for policy and examine the degree to which mathematical models meet these standards. These criteria are derived from the four fundamental principles of biomedical ethics (5): *autonomy* (i.e., the right to make informed decisions, which in turn requires sufficient *independent* and *transparent* information), *beneficence* (i.e., being of benefit to the end user), *non-maleficence* (i.e., doing no harm), and *justice* (i.e., fair distribution of benefits, risks, and costs). The principles of independence and transparency are also articulated specifically in case law as the necessary foundation for scientific advice, which informs risk assessment (6, 7). The latter are fundamental precursors for autonomy but were thought best considered as distinct and separate criteria. Conversely, beneficence and non-maleficence are condensed into a single criterion (see Table 1 for a summary).

**Table 1:** **A framework for modelers to promote ethical “good practice” in building a scientific evidence base for policy development**.

Criteria	Summary description	Evaluation of ethical risk
Independence	Models are subjective tools, which reflect modelers’ perception of the system at risk. They are influenced by prior art in the area and cultural research links between individuals/institutes. Independence is compromised if conflicts of interest arise (e.g., if modelers are responsible for both design and communication of model merits, as increasingly is the case)	Is the model provenance known and well documented (i.e., the full history of model development, including funding sources, conceptual design, coding, verification, peer-review processes and publication, as well as the modelers involved in the development)? Has the model been validated using independent data sources not used for model parameterization?

Transparency	A high level of technical skill and expertise and some fluency in the language of mathematical models are required to communicate model constraints, uncertainties, and assumptions to policymakers. This makes it difficult for complex models to be scrutinized by a diversity of relevant audiences. Without the assistance of experts in risk communication who can broker this knowledge, and robust frameworks for knowledge exchange, policymakers may misinterpret strengths and weakness of model recommendations	Is there clear documentation of the scientific approach so that methods are robust, repeatable and reproducible? Is there information about potential conflicts of interest, constraints, or biases affecting data collection and analyses (e.g., racial, ethnic, class, sexual, and gender issues) and any assumptions or uncertainties inherent in the modeling process? Is the model code open source or available on request? Are the model assumptions well described and documented and understood by policymakers?

Beneficence*/*non-maleficence	Beneficence is contingent on excellent, ethical model design and diligent protection of data subjects. Irreducible model uncertainties may inadvertently expose research subjects/stakeholders to risks without a guarantee of beneficial outcomes for the population. Few models are evaluated to determine whether recommendations are accurate or effective because there are few “checks and balances” for post-dissemination model quality or utility.	If a policy decision is based on the model evidence, is it more likely to be effective and beneficial than a decision made in the absence of the model? Has the model been verified, i.e., does it do what the modeler wants it to do? Has the model been validated? (i.e., does it realistically map onto what is occurring in real life). What are the sources and magnitude of uncertainty in the model—are these associated with parametric uncertainty or model selection?

Justice	Models are useful “thought experiments.” However, if model evidence is intended to inform policy in the real world, modelers have a duty of care to consider and communicate ethical issues. Ethical risks are influenced by model variability and uncertainty that have important impacts on the distribution of beneficial or harmful consequences.	Is any lack of knowledge about important parameters attributable to uncertainty or variability? Where possible, is model variability attributed to known factors, to create more ethical outcomes? If interventions based on model predictions are implemented in the real world, can the predicted benefits and harms to different individuals and subpopulations be quantified?

### Independence

Scientific evidence should be derived autonomously through objective, established methods and subject to scientific and ethical review, to avoid “the misuses or abuses of … information for political purposes or inefficient regulation due to overestimation of scientific sources” [(8), p. 147]. Conflicts of interest must be identified and resolved so that actors at the science–policy interface have clear lines of accountability for the consequences of their decisions.

Models may be developed independently, but are likely to be influenced by direct or indirect dialogue with policymakers, which may create unintended opportunities for them to become tools for policy-based evidence making, rather than evidence-based policymaking (9). Although mathematics is often assumed to be “free of ideological or ethical issues” [(10), p. 2], model frameworks are typically informed by how modelers perceive the system at risk, translate knowledge into quantitative parameters (11, 12), prioritize inclusion or exclusion of different drivers of influence and transparently compose, and transcribe and share the mathematical code and data for the construction of a model framework (10). Specific modeling approaches have cultural histories associated with individuals, institutions, and preferences of funding bodies that heavily influence and impact upon model framework selection, subsequent model evaluation, and their ability to be objectively critiqued (13). Consensus between multiple, seemingly “independent” models may not be meaningful if data sources and cultural history are shared between modeling groups (14).

### Transparency

Transparency refers to the clear documentation of the scientific approach so that methods are robust, repeatable, and reproducible, and outcomes are clearly communicated and understood. This documentation includes information about conflicts of interest, constraints, or biases affecting data collection and interpretation (e.g., intersectional analysis of the effect of class, racial, ethnic, gender, or sexual categorizations) and any assumptions or uncertainties inherent in the modeling process.

Mathematical models are documented and communicated through physical representation in text, tables, or figures and through formal discussion within and between the scientific community, decision-makers, and the public [(14), p. 2]. In academia, model coding practices do not conform to industrial standards and are not always open source, making them difficult to reproduce, compare, or independently critique. Model parameters are typically estimated by statistical inference or expert opinion and may be extrapolated from comparable (to a greater or lesser extent) data if there is scientific uncertainty or a weak evidence base for a system (15). When data are scarce, assumptions about important “drivers” (i.e., risk factors) in the model will become more influential. However, if multiple, interdependent assumptions about immeasurable uncertainties are made, it is difficult to meaningfully quantify the impact of these upon the credibility and accuracy of model results. This is exacerbated if modelers are reluctant to express model uncertainty in case it undermines the credibility of the model in the eyes of the model user (14). Ambiguity over model assumptions and methods, variability and uncertainty, and matters of ethics and equity of outcomes may be difficult for decision-makers to identify, if risk is communicated as a single estimate [(16), p. 310]. Transparency is further impaired because of the lack of a common language between modelers and policymakers, where the latter will have limited knowledge of the specialist mathematical and statistical techniques utilized. In the absence of other contextualizing evidence, models and algorithms can become designated as a “black box,” creating a powerful “regime of justification,” which is unlikely to be questioned or held to account (ethically or empirically) by end-users [(10), p. 12] who may have a reputational commitment to favored projects and an unrealistic picture of the certainty associated with the system. This idea has been conceptualized as an intermediate “trough of certainty” between practitioners, cognizant with methodological limitations and uncertainties, and those far removed (e.g., the public), who have little belief in such expert opinion (17). Consequently, policymakers may incorrectly hold social, political, or economic factors more accountable than model failures for any undesirable outcomes.

Alternative methods, such as multicriteria decision analysis, have been adopted in some disciplines to support transparency in benefit-risk assessment (18). Similarly, mathematical modeling could be considered a convenient framework to integrate and interpret different types of evidence, while trying to capture aspects of the “true” reality.

### Beneficence/Non-Maleficence

Research design (including choice and allocation of any interventions) should be sound, protect, and prevent subjects from experiencing harm (i.e., adhere to principles of non-maleficence) and provide accurate, precise answers that are robustly derived, recorded, and reported by competent experts in relevant fields. Beneficent research should not only be excellent in design but also aim to enhance welfare for participants and (by extension) stakeholders. Scientifically invalid research is always unethical because it exposes research subjects to risks without possible benefit [(3), p. 7].

Access to robust, reliable, relevant, and timely data is one of the biggest obstacles to achieving model beneficence. Researchers must balance their desire for wider data access and sharing with their responsibility to ensure that data subjects are not exposed to (non-physical) harms. Risks to individuals’ rights to privacy and issues of confidentiality and consent have emerged with new data mining and statistical methodologies that facilitate greater exploitation of “big” data sets. Although historically management of data quality, lifecycle, and security may have been undertaken in a relatively *ad hoc* manner by some individual researchers or institutions, increased opportunities and requirements for data sharing in combination with new, more stringent data legislation and examples from case law (19) indicate that greater emphasis must be placed on auditable data management processes (20, 21).

Even if data are available, there will always be uncertainty in model choice and parameterization, as well as unknown uncertainties in the system that cannot be empirically measured or tested and thus never fully accounted for in any model framework. For example, useful predictive mathematical models are unlikely to perfectly reconstruct past epidemic events (4, 14), defining an implicit “trade-off” between the definition of observed “noise” as stochastic (random) variability or as unknown uncertainty. This is particularly problematic in models that are developed to predict the occurrence and trajectory of rare events when data are scarce and the timeframe for prediction is long.

The degree of model uncertainty has important impacts on model beneficence. This was illustrated in 2014, when models were published providing imprecise range estimates of the number of predicted Ebola infections (22). Although there are arguably benefits to preparing for “worst-case” scenarios, as in this case, there are also important practical (i.e., inappropriate investment in, and allocation of, health resources) and intangible costs (i.e., loss of credibility and trust) from imprecise predictions, especially if the model uncertainties are poorly communicated. Uncertainty in model selection has the potential to generate both uncertainty and bias (i.e., systematic errors). Models in which uncertainty about the outcome is associated with parameter uncertainty or stochastic variability may be less prone to bias. A model, which estimates a wide interval in which future observations/events may fall, can be seen to be beneficent if that interval includes the ultimate observed outcome, while genuinely reflecting the degree of uncertainty around the outcome.

Policy decisions based on model evidence should be more effective than decisions made in the absence of a model (1). An excellent, beneficent model is one that does what the modeler wants it to do (verified) but also realistically maps to what is occurring in real life (validated) (1, 4). Model evaluation (validation and verification) is important as it offers some quality assurance on model utility. Most models are verified, but fewer are fully validated because of the lack of harmonized standards within or between research groups, and a dearth of timely data for this purpose (23, 24). As a result, model evaluation often relies on the same sparse data used to initially parameterize the model. Sensitivity analyses (which vary parameter values to quantify the impact of uncertainty) are offered as supporting evidence to demonstrate model robustness. However, these may be of limited value if the parameter being analyzed is not well specified in the model because there is no evidence base at all. Furthermore, as all sensitivity analyses are carried out within the reference frame of the chosen model, it tells the user nothing about the interplay between the parameter and model choice uncertainty (15). Scientific consensus about model outputs is also utilized to increase end-user confidence in model outputs (and highlight model excellence). However, consensus is not improbable if a narrow range of policy options is explored and different modeling groups have access to the same data, share a common modeling culture and funding sources, and seek to publish in similar journals with the same pool of reviewers.

Once produced, mathematical models are easy to implement and can become tools to support “fast-track” decision-making. This may be desirable for decision-makers but is paradoxically less useful in early stages of disease outbreaks that are often characterized by “great chaos and uncertainty” (25). Unlike pharmaceuticals (26) or medical devices [(27), (28), Art 1, (29)], there are few, if any, regulatory procedures that provide checks and balances about model quality or utility, and which would prevent inaccurate or ineffective modeling results from being rapidly, and perhaps imprudently, translated and disseminated to policymakers or the public. Specifically, there are no mechanisms for explicit *post hoc* evaluation of model effectiveness, which would provide feedback to practitioners and close the science-policy loop. Once a model is widely disseminated and utilized, its evidence may tend to become increasingly impervious to future challenges over legitimacy (10).

### Justice

Scientists have an obligation to consider the equitable and non-discriminatory distribution of benefits and burdens of research in light of the risks that may be undertaken by individuals in the population (3). Researchers cannot be held accountable for existing inequalities in populations at risk, but they must not worsen or create new inequalities or unfair consequences (3). Thus, in assessments of risk, scientists need to facilitate consideration of not only the likelihood of a hazard occurring but also the consequences of its occurrence and/or the opportunity costs of managing it through control strategies applying to various subpopulations.

The use of forecasting models as decision support tools enables premeditated rather than reactive decisions about how, when, and to whom interventions will be applied to minimize harm. Modelers identify in advance how interventions are “best” allocated and thus determine who is likely to experience resultant benefits or harms if these are implemented in real-life scenarios. The consequences of unfair distribution of benefits and harms are non-trivial to incorporate into quantitative approximations of risk and thus are often excluded from interpretations of model analyses. For example, avian influenza models propose widespread culling of susceptible poultry and mass administration of antiviral drugs in combination with regional quarantine to maximize overall public benefit for the international community by containing disease at its source (30). Although this utilitarian approach is scientifically sensible, it will invariably result in new inequalities regarding access to antiviral medication, exposure to risk, and imposition of restrictions on the rights and freedoms of certain individuals within disease-affected communities. Furthermore, these burdens are likely to be disproportionately borne by vulnerable individuals in low-income countries where disease is endemic and other risk factors for disease may predominate.

Inequalities in benefits or harms experienced within populations occur due to population variability. Variability is either attributable to individual heterogeneity or differences in the population due to other exogenous factors (e.g., wealth, education, nutrition, sanitation, available healthcare, geographical location). Depending on the viewpoint of the decision-maker, the complex nature of the population “at risk” might be thought of as either variability or uncertainty. A hypothetical example is the use of a mathematical model to evaluate the efficacy of an intervention with different impacts on individuals with different genetic properties. Individuals might choose to see a lack of knowledge about their specific situation as uncertainty, which could in principle be resolved in the model to give a more “personal” range of outcomes. Given knowledge of one’s own genetics, it could be possible to use the model to judge whether the intervention is personally advantageous or not. A policymaker might choose to see heterogeneity as variability, which can be averaged across the population, when deciding whether the intervention should be approved for use across the health services. A reliance on only such averages may be unethical. If the output from a model does not facilitate consideration of the effect of interventions on individuals, or at least identifiable subpopulations, then, since potential ethical risks are foreseeable, but unresolved, the model framework is unlikely to meet the “justice” test. Where variability can be attributed to known factors, provided that relevant parameters are known, it should be accounted for in the model design to reduce overall inequality and avoid unethical and unfair outcomes where possible (31). Differing perspectives on the availability and reliability of data might lead to different assessments of the extent to which a model meets the “justice” criterion. The design of models, the treatment of data, and the approach to interpreting results have considerable bearing on whether policy decisions are effective and ethical in practice.

## Translation from Science to Policy: Accountability

*Modeling* risks (and risk reduction) and *managing* risks are arguably two separate, independent activities. The former is the responsibility domain of the scientist; the latter belongs to the policymaker. However, the communication of advice on risk connects science to policy and blurs this distinction.

Ultimately, scientists have a responsibility to ensure that policymakers understand the strengths and weaknesses of the scientific evidence base. This includes an understanding of the socioeconomic and ethical impacts of biased or uncertain predictions that may lead to inappropriate policy implementation. Inaccurate model forecasts have rarely been punished: scientists in universities and non-governmental organizations do not have a “duty of care” and are not personally exposed to liability (26, 32). However, there is a growing public demand for accountability in delivery of effective and accurate risk communication from scientists and policymakers; this trend may have legal consequences in the future [(33), (34), p. 16, (35)].

Mathematical models, as stand-alone predictive risk assessment tools, have infrequently met all of the criteria for an ethical scientific evidence base for policy development. However, this does not preclude models from being useful tools for risk assessment, provided they are integrated with other types of qualitative and quantitative evidence. Mathematical models may be most ethical and best utilized in an exploratory context, as one of several complementary sources of scientific evidence that underpin strategic decisions about disease contingency planning.

To operationalize mathematical models so that they provide useful evidence for policy purposes, the following initiatives might be considered:

1. *Creation of partnerships between scientists, funding bodies, policymakers, and public stakeholders* to mutually define the range of questions legitimately answerable *via* modeling, transparently communicate model outputs, and support interpretation of results and *post hoc* evaluation of the effectiveness of model recommendations as implemented. Models should be developed and assessed using interdisciplinary, reflexive approaches that incorporate stakeholder knowledge and include agreed values and interests to promote trust and compliance with resulting model strategies.
2. *Development and progressive international harmonization of minimum standards of acceptable practice in model development, parameterization, evaluation, and reporting*. Coding standards and guidelines of best practice should be widely adopted, and the remit of the peer-review process, as it applies to the academic mathematical modeling literature, extended to incorporate concepts relating to transparency, beneficence, independence, and justice. Current peer-review practices are likely to be effective in handling issues relating to transparency and beneficence. Issues of independence and justice may require a greater cultural shift in peer-review practices if their explicit inclusion in the review exercise is to prove worthwhile.
3. *Universal registration of mathematical models utilized as evidence in policymaking*. A repository of published and unpublished mathematical models [analogous to the Cochrane Library (36)] would help support an efficient, collaborative international approach to contingency planning for animal and human disease risks. This approach, which would create a transparent, searchable archive of evidence, could be a first step toward greater accessibility of model code (either directly *via* provision of open-source model code or indirectly *via* provision of pseudocode).
4. *Auditable processes for data stewardship* should be a component of every modeling study to protect stakeholders’ rights to privacy and confidentiality according to current legislation and ethical guidelines. Data stewardship includes responsibility for the administration, quality assessment, management, and security of all data and metadata.
5. *Bespoke ethical advisory bodies* should be created to advise modelers of potential ethical risks and oversee the translation of modeling advice into policy. These bodies could subsequently act as knowledge brokers across the science–policy interface to facilitate ethical risk communication.

## Conclusion

Models have a useful and important role in informing policy, provided they are created robustly and ethically. This study is part of a larger programme of work assessing model resiliency and utility for outbreak preparedness, in which we are exploring whether inclusion of an ethical impact statement based on this framework, will deliver benefits to policymakers who are relying on model evidence in their decision-making processes. Modelers cannot absolve themselves of ethical responsibilities arising in model interpretation and implementation but have the option to create interdisciplinary collaborations with policymakers, stakeholders, and other experts to ensure these obligations are fulfilled. Ethical models will ultimately inspire and inform ethical policy choices.

## Ethics Statement

The work presented in this manuscript did not involve human or animal subjects.

## Author Contributions

LB was responsible for the concept, design, and writing of the manuscript. IM made substantial contributions to the design, content, and critical revision of the manuscript. Both authors are accountable for the accuracy and integrity of this work and have approved the final manuscript for publication.

## Conflict of Interest Statement

None of the authors of this paper have a financial or personal relationship with other people or organizations which could inappropriately influence or bias the content of the paper.
